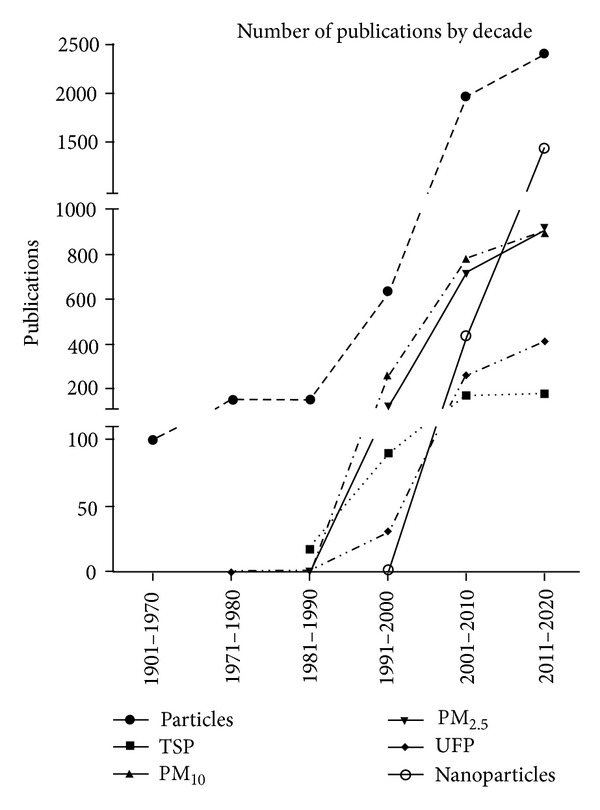# Particulate Matter and Nanoparticles Toxicology

**DOI:** 10.1155/2013/642974

**Published:** 2013-07-10

**Authors:** Ernesto Alfaro-Moreno, Tim S. Nawrot, Abderrahim Nemmar, Irma Rosas, Per Schwarze

**Affiliations:** ^1^Environmental Toxicology Laboratory, Instituto Nacional de Cancerología, Avenida San Fernando 22, Tlalpan, 14080 México City, CP, DF, Mexico; ^2^Centre for Environmental Sciences, Hasselt University, 3500 Hasselt, Belgium; ^3^Department of Public Health, Leuven University (KU Leuven), 3000 Leuven, Belgium; ^4^Department of Physiology, College of Medicine and Health Sciences, United Arab Emirates University, Al Ain 17666, UAE; ^5^Aerobiology Laboratory, Atmospheric Sciences Center, Universidad Nacional Autónoma de México, 04510 México City, DF, Mexico; ^6^Department of Air Pollution and Noise, Norwegian Institute of Public Health, 0403 Oslo, Norway

Humans have been exposed for thousands of years to particulate matter (PM) from natural and anthropogenic sources. Since the first third of the twentieth century, health problems related to dust exposure in miners have been documented [1]. Early epidemiological data have shown evidence of the relation between inhalation of PM and several lung diseases, including lung fibrosis and lung cancer [2]. The Meuse valley fog of 1930 [3], the Donora smog incident of 1948 [4], and the London great smog event of 1952 [5] were the foundation to create a legislation regarding to air pollutants. In the US, the Clean Air Act was enacted in 1972. Before 1970, the main efforts in this field were aimed at measuring the PM suspended in the air and its relation to death increases associated to lung diseases. During the 1970s and 1980s, the measurements of environmental particles improved, and efforts to quantify particles with different aerodynamic sizes gave a view on how the particles could be related to different diseases. These methods helped to identify sources of different particles, leading to specific actions to control the emission of PM. During the 1990s and the first decade of this century, a great effort has been done to determine the cellular and molecular mechanisms related to the particle toxicity, and many studies have shown that particle size and composition play central roles in the biological effects. During the last 20 years, a great concern has grown regarding the use of nanoparticles (NPs) and its possible impact on workers and final users. To present, it is evident that inhaled particles may have local and systemic effects, and that the size, the composition, and the physicochemical characteristics of these airborne particles play a central role in their toxicity. 

Despite all the clinical, epidemiological, and toxicological evidence, we are far from understanding the toxicology of particles, in part by the combined effects and interactions of various substances mixed within the particles. In addition, the lack of evidence of a threshold value makes it difficult to set safe limit values. Therefore, a constant growth in the total number of publications related to urban PM and NP is easy to observe when a simple search is done on PUBMED. When the words “particles” and “air pollution” are searched, 101 publications are found from 1900 to 1970. The number rises to 149 from 1971 to 1980. During the 1980s, the first efforts were done to evaluate the effects of particles with different aerodynamical sizes but the number of publications remained in 150. Later on, during the 1990s, the evaluation of particles with different aerodynamic sizes (PM_10_, PM_2.5_, and ultrafine particles) made the number of publications to grow up to more than 600. The continuous evaluation of urban particles and the arising use of nanomaterials led to almost 2000 publications during the first decade of this century. If the rate of publications on these fields keeps the same rhythm as the first two years of the present decade, we will find about 2400 publications for the 2011–2020 decade (Figure 1).

The previously-mentioned numbers gave a clear idea of why a special issue on particulate matter and nanoparticles toxicology is important for this field. In this special issue, we are publishing a selection of studies dealing with particle sampling and characterization, *in vitro* toxic effects characterization using traditional and novel models, *in vivo* effects of PM, and *in vivo* and *in vitro* effects of different types of NP. 

We also include three reviews, discussing the cellular effects of diesel particles, another one discussing the evidence relating the exposure to particles and other inhaled pollutants to the increased risk of Alzheimer and Parkinson's diseases. Finally, a review of the state of the art in the *in vivo* and *in vitro* toxicological characterization of particles was prepared for this issue by the guest editors, where we discuss the latest evidence of local and systemic effects induced by inhaled particles.

Great efforts have been done during the last 50 years to understand the toxicology of particulate matter, and much information is available helping to understand the risks of exposure to different types of particles. Nevertheless, there is much to do in the field, and the efforts presented here will be of great value to push further the frontiers of our knowledge on the particulate matter toxicology field.

*Abderrahim Nemmar* *Ernesto Alfaro-Moreno* *Irma Rosas* *Per Schwarze* *Tim S. Nawrot*

## Figures and Tables

**Figure 1:**